# Total cholesterol and mortality in peritoneal dialysis: a retrospective cohort study

**DOI:** 10.1186/s12882-023-03187-1

**Published:** 2023-05-23

**Authors:** Junnan Wu, Ruifeng Yang, Xiaoyang Wang, Xiaojiang Zhan, Yueqiang Wen, Xiaoran Feng, Niansong Wang, Fenfen Peng, Guihua Jian, Xianfeng Wu

**Affiliations:** 1 Department of Nephrology, Zhejiang University Medical College Affiliated Sir Run Run Shaw Hospital, HangZhou, China; 2 Department of Nephrology, Shanghai Sixth People’s Hospital, Shanghai Jiao Tong University School of Medicine, No.600, Yi Shan Road, Shanghai, 200233 China; 3 Department of Nephrology, The First Affiliated Hospital of Zhengzhou University, Zhengzhou, China; 4 Department of Nephrology, The First Affiliated Hospital of Nanchang University, Nanchang, China; 5 Department of Nephrology, The Second Affiliated Hospital of Guangzhou Medical University, Guangzhou, China; 6 Department of Nephrology, Jiujiang No. 1 People’s Hospital, Jiujiang, China; 7 Clinical Research Center for Chronic Kidney Disease, Shanghai Sixth People’s Hospital, Shanghai Jiao Tong University School of Medicine, Shanghai, China; 8 Department of Nephrology, Zhujiang Hospital of Southern Medical University, Guangzhou, China

**Keywords:** Total cholesterol, Peritoneal dialysis, Mortality

## Abstract

**Background:**

Total cholesterol is inversely associated with mortality in dialysis patients, which seems implausible in real-world clinical practice. May there be an optimal range of total cholesterol associated with a lower mortality risk? We aimed to evaluate the optimal range for peritoneal dialysis (PD) patients.

**Methods:**

We conducted a retrospective real-world cohort study of 3565 incident PD patients from five PD centers between January 1, 2005, and May 31, 2020. Baseline variables were collected within one week before the start of PD. The associations between total cholesterol and mortality were examined using cause-specific hazard models.

**Results:**

820 (23.0%) patients died, including 415 cardiovascular deaths, during the follow-up period. Restricted spline plots showed a U-curved association of total cholesterol with mortality. Compared with the reference range (4.10–4.50 mmol/L), high levels of total cholesterol (> 4.50 mmol/L) were associated with increased risks of all-cause (hazard ratio [HR] 1.35, 95% confidence index [CI] 1.08–1.67) and cardiovascular mortality (HR 1.38, 95% CI 1.09–1.87). Similarly, compared with the reference range, low levels of total cholesterol (< 4.10mmol/L) were also associated with high risks of all-cause (HR 1.62, 95% CI 1.31–1.95) and cardiovascular mortality (HR 1.72, 95% CI 1.27–2.34).

**Conclusion:**

Total cholesterol levels at the start of PD between 4.10 and 4.50 mmol/L (158.5 to 174.0 mg/dL), an optimal range, were associated with lower risks of death than higher or lower levels, resulting in a U-shaped association.

**Supplementary Information:**

The online version contains supplementary material available at 10.1186/s12882-023-03187-1.

## Background

Cholesterol is essential for various cellular processes and is also involved in the pathogenesis of cardiovascular disease [1, 2]. Hypercholesterolemia is an important risk factor for atherosclerotic progression and long-term mortality in the general population [3]. However, data from large cohort studies and clinical trials suggest that cardiovascular disease prevention and treatment guidelines cannot be transferred uncritically from individuals with intact renal function to dialysis patients. Serum lipid levels are often abnormal in dialysis patients. As an example, patients on peritoneal dialysis (PD) have higher serum levels of total cholesterol and low-density lipoprotein cholesterol (LDL-C) [4].

Dialysis patients are at increased risk of death [5]. Many risk factors for death appeared to have the opposite effect on outcomes compared to what was observed in the general population. This inverse association between risk factors and mortality was particularly pronounced for cholesterol levels. Several studies have shown that low cholesterol is associated with poor outcomes in end stage renal disease (ESRD) subjects, whereas hypercholesterolemia appears to be protective [6–9]. The 2014 KDIGO guidelines for lipid management recommend that statins should not be used in dialysis-dependent patients [10], because malnutrition-related syndromes may adversely affect mortality [8]. Nonetheless, the inverse association seems implausible in real-world clinical practice. Too lower or higher cholesterol associated with a higher mortality risk seems more plausible in clinics. Therefore, may there be an optimal range of total cholesterol associated with lower mortality risk in dialysis patients? We hypothesized a U-curved association between total cholesterol and mortality in dialysis patients. In the present study, we aimed to examined the optimal range of total cholesterol associated with the lowest riks of mortality in patients on continuous ambulatory peritoneal dialysis (CAPD) in a real-world setting.

## Materials and methods

### Study design and patients

We conducted a retrospective real-world cohort study that included 3565 incident Chinese CAPD patients from five PD centers between January 1, 2005, and May 31, 2020. To maximumly represent the real-world setting of the CAPD population, no patient was excluded from this study. The requirement for informed consent was waived by the Ethics Committee of (The First Affiliated Hospital of Nanchang University, Nanchang, China. The First Affiliated Hospital of Zhengzhou University, Zhengzhou, China. Jiujiang No. 1 People’s Hospital, Jiujiang, China. Zhujiang Hospital of Southern Medical University, Guangzhou, China. The Second Affiliated Hospital of Guangzhou Medical University, Guangzhou, China**)** because of the retrospective nature of the study. The study protocol complied with the Declaration of Helsinki and had full approval from each Clinical Research Ethics Committee.

### Follow up and data collection

We respectively collected demographic data, comorbidities, medication use, and laboratory variables one week (5.3 ± 1.2 days) before the start of PD, including age at study entry, sex, body mass index, current smoker, current alcohol use, systolic blood pressure, diabetes mellitus, prior cardiovascular disease, hypertension, beta-blockers, angiotensin-converting enzyme inhibitors/angiotensin II receptor blockers (ACEI/ ARB), diuretics, statins, albumin, estimated glomerular filtration rate (eGFR), total cholesterol, high-density lipoprotein cholesterol (HDL-C), and LDL-C.

Our primary and secondary endpoints were all-cause and cardiovascular mortality, respectively. Details for the CAPD follow-up were previously described elsewhere [11]. The follow-up period was from the start of PD to the date of death, transfer to hemodialysis, receiving renal transplantation, transfer to other dialysis centers, loss of follow-up, or May 31, 2020. Patients lost to follow-up were censored at the date of the last examination.

### Statistical analysis

Differences in the baseline characteristics stratified by total cholesterol were compared using the chi-square test for categorical variables and analysis of variance for continuous variables. Restricted-cubic-spline plots were used to explore the shape of the association between total cholesterol and mortality, fitting a restricted-cubic-spline function with four knots (at the 25th, 50th, 75th, and 95th percentiles) [12].

Based on our restricted-cubic-spline plots for the primary endpoint, we selected a level of 4.10 to 4.50 mmol/L as the reference category for total cholesterol. To explore the association of total cholesterol with mortality, we primarily used cause-specific hazard models. We then constructed sub-distribution hazard models to confirm the association observed in the primary analysis. All factors were included in the multivariate analysis based on their clinical significance. Transfer to hemodialysis, receiving renal transplantation, transfer to other centers or loss of follow-up was considered a competing risk. The main difference between the two hazard models is that subjects experiencing a competing risk event remain in the risk set in the sub-distribution hazard model. In contrast, they are removed in the cause-specific hazard model [13, 14]. We tested for interactions of age, sex, diabetes mellitus, prior cardiovascular disease, hypertension, and malnutrition (albumin < 36.0 g/L).

### Sensitivity analysis

To minimize the potential for reverse causation, we conducted analyses that excluded patients with prior cardiovascular disease or those deaths in the first 2 years of follow-up. As for those patients with a short-term follow-up period, the interesting endpoints may not be observed, under-estimating the incidence of mortality. We further analyzed the association in patients with at least 24 months of follow-up for fully observing endpoints. In addition, to minimize the effect of beta-blockers, diuretics, or statins on total cholesterol, we also analyzed the association in patients without beta-blockers, diuretics, or statin use. Missing data for total cholesterol (n = 35) or any other explanatory variables (n = 121) at the start of PD were replaced by the most recent available values by checking patients’ medical records at the first PD procedure. All analyses were conducted with the use of Stata 15.1. statistical software (StataCorp, College Station, TX).

## Results

### Baseline characteristics

**Fig. 1:**
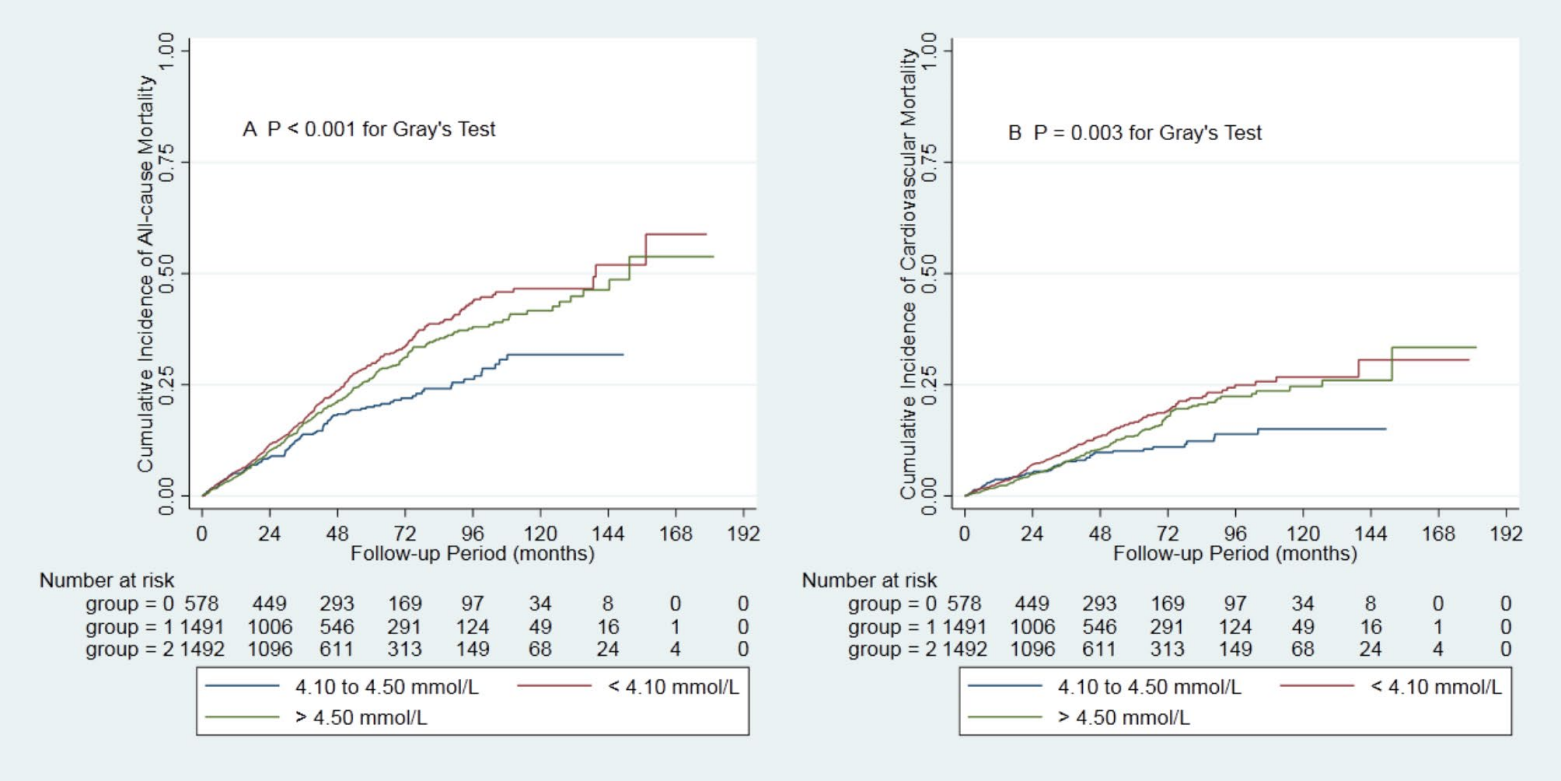
Association of total cholesterol with risk of mortality Panel A showed a restricted-cubic-spline plot of the association between total cholesterol and all-cause mortality. Panel B showed a restricted cubic-spline plot of the association between total cholesterol and cardiovascular mortality. All plots were adjusted for age, sex, body mass index, current smoker, current alcohol use, systolic blood pressure, comorbidities, medication use, and laboratory variables. Dashed lines indicate 95% confidence intervals. The median total cholesterol (4.30 mmol/L) was the reference standard, indicated by the grayline

Of 3565 patients, the median age was 49.0 (38.0–60.0) years, and 52.1% of patients were male sex. The median total cholesterol levels were 4.38 ± 1.19 mmol/L. Based on restricted cubic spline plots for all-cause mortality, we selected a level of 4.10 to 4.50 mmol/L as the reference category for total cholesterol (Fig. 1). Table 1 presented the characteristics of patients by categories of baseline total cholesterol. Patients with high total cholesterol levels were more likely to be current smokers, diabetes mellitus, prior cardiovascular disease, ACEI/ARB, diuretics, higher systolic blood pressure, eGFR, HDL-C, and LDL-C.

**Table 1:** Baseline characteristics by categories of total cholesterol

	Total cholesterol			
	Low (< 4.10 mmol/L)	Moderate (4.10 to 4.50 mmol/L)	High (> 4.50 mmol/L)	P-value
Number of patients, n	1492	579	1494	
Cholesterol (mmol/L)	3.34 ± 0.54	4.30 ± 0.12	5.44 ± 0.92	< 0.001
Age (years)	48.5 ± 15.1	49.3 ± 15.3	49.8 ± 15.0	0.079
Male sex, n (%)	795 (53.28%)	314 (54.23%)	747 (50.00%)	0.104
Body mass index (kg/m^2^)	22.2 ± 3.3	22.2 ± 3.1	22.4 ± 3.3	0.262
Current smoker, n (%)	186 (12.47%)	46 (7.94%)	122 (8.17%)	< 0.001
Current alcohol use, n (%)	68 (4.56%)	16 (2.76%)	45 (3.01%)	0.038
Systolic blood pressure (mmHg)	135.2 ± 22.4	137.0 ± 21.8	139.6 ± 23.4	< 0.001
Comorbidities, n (%)				
Diabetes mellitus	219 (14.68%)	110 (19.00%)	345 (23.09%)	< 0.001
Prior cardiovascular disease	124 (8.31%)	59 (10.19%)	196 (13.12%)	< 0.001
Hypertension	1030 (69.03%)	397 (68.57%)	1042 (69.75%)	0.847
Medication use, n (%)				
Beta-blocker	525 (35.2%)	234 (40.4%)	579 (38.8%)	0.039
ACEI/ARB	373 (25.0%)	157 (27.1%)	436 (29.2%)	0.037
Diuretics	200 (13.40%)	103 (17.79%)	254 (17.00%)	0.008
Statin	225 (15.08%)	69 (11.92%)	230 (15.39%)	0.115
Laboratory measurements				
Albumin (g/L)	34.7 ± 5.4	34.5 ± 5.3	34.3 ± 5.3	0.093
eGFR (mL/min*1.73m^2^)	6.91 ± 3.86	6.96 ± 3.55	7.56 ± 3.89	< 0.001
HDL-C (mmol/L)	1.02 ± 0.33	1.19 ± 0.35	1.23 ± 0.45	< 0.001
LDL-C (mmol/L)	2.40 ± 0.86	2.55 ± 0.66	2.72 ± 0.96	< 0.001

ACEI/ARB, angiotensin-converting enzyme inhibitor/angiotensin receptor blocker; eGFR, estimated glomerular filtration rate; HDL-C, high-density lipoprotein cholesterol; LDL-C, low-density lipoprotein cholesterol

### Total cholesterol and endpoints

**Fig. 2:**
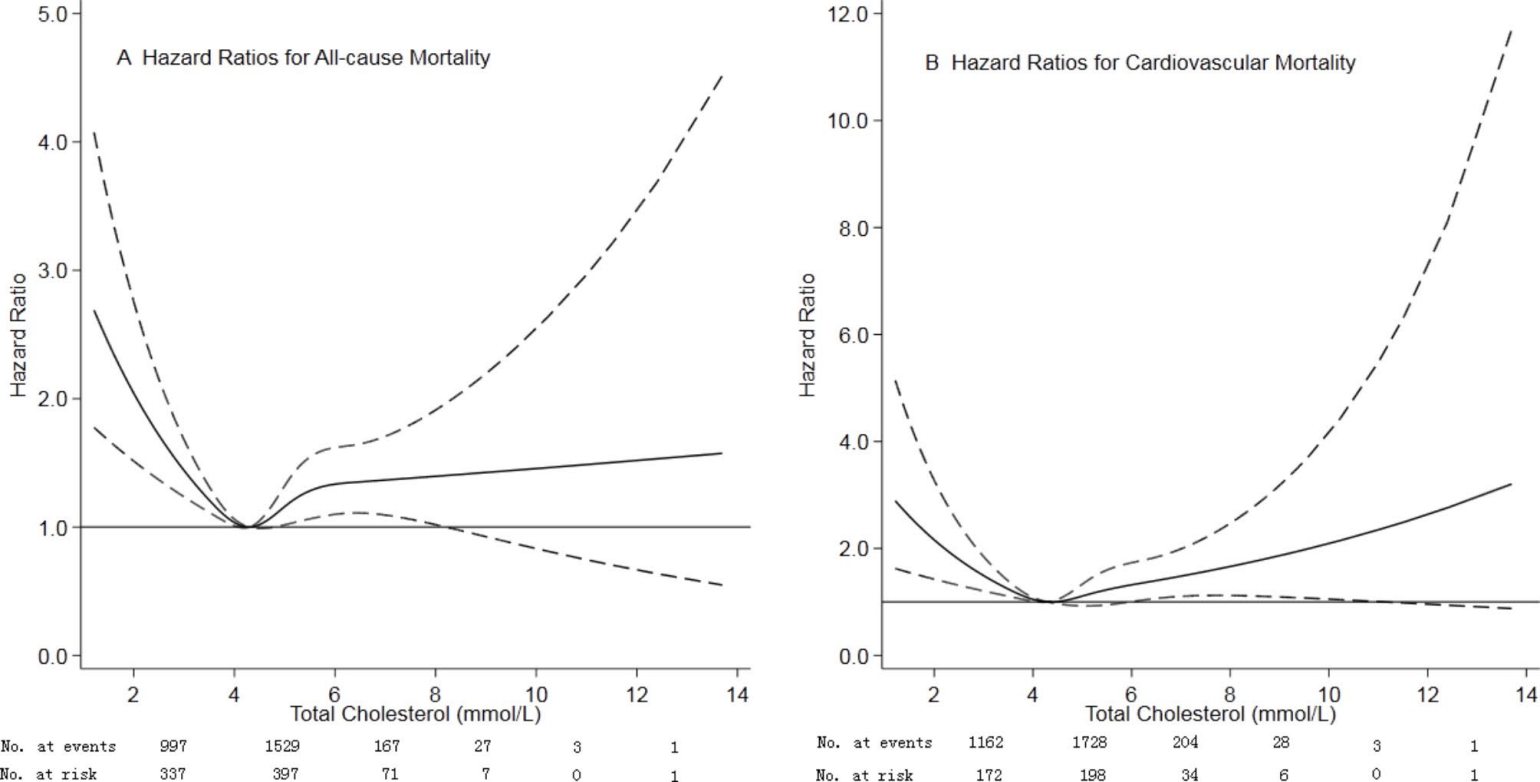
Cumulative mortality by categories of total cholesterol Panel A showed cumulative all-cause mortality by categories of total cholesterol. Panel B showed cumulative cardiovascular mortality by categories of total cholesterol

During the 14131.6 person-years of follow-up, 820 (23.0%) patients died, 481 (13.5%) patients transferred to hemodialysis, 241 (6.8%) patients received renal transplantation, 459 (12.9%) patients transferred to other dialysis centers, and 61 (1.7%) patients had been the loss of follow-up. Of 820 deaths, 415 (50.6%) died of cardiovascular disease, 142 (17.3%) died of infectious disease, 76 (9.3%) died of gastrointestinal bleeding, 15 (1.8%) died of malignancy, 172 (21.0%) died of other reasons. Deaths occurred in 363 (65.7/1000 person-years), 111 (42.1/1000 person-years), and 346 (58.0/1000 person-years) patients in those < 4.10, 4.10–4.50, and > 4.50 mmol/L patients, respectively (Table 2). Cumulative all-cause and cardiovascular mortality were significantly lower in the moderate group (crude analysis, P < 0.001, Fig. 2; multivariate analysis, Figure S1).

**Table 2:** Incidence rate of death according to total cholesterol*

	Total cholesterol			
	All levels	Low (< 4.10 mmol/L)	(Moderate 4.10 to 4.50 mmol/L)	High (> 4.50 mmol/L)
All-cause mortality				
Deaths, n	820	363	111	346
Deaths, per 1000 person-years	58.0	65.7	42.1	58.0
Cardiovascular mortality				
Deaths, n	415	190	54	171
Deaths, per 1000 person-years	29.4	34.4	20.5	28.7

*The incidence rate was calculated by dividing the proportion of events by the total effective observation time in the risk, which is converted to the number of episodes per 1000 years

In the multivariate cause-specific hazard model, compared with the moderate group, the adjusted hazard ratios (HRs) of all-cause mortality were 1.62 (95% CI, 1.31 to 2.01) and 1.35 (95%CI, 1.08 to 1.67) for the low and high group, and the adjusted HRs of cardiovascular mortality were 1.72 (95% CI, 1.27 to 2.34) and 1.38 (95%CI, 1.09 to 1.87) for the two groups, respectively (the multivariate model in Table 3).

**Table 3:** Association between total cholesterol and all-cause mortality*

	HR (95% CI) by total cholesterol		
	Low (< 4.10 mmol/L)	(Moderate 4.10 to 4.50 mmol/L)	High (> 4.50 mmol/L)
Univariate model	1.58 (1.27 to 1.95)	1.0	1.39 (1.12 to 1.72)
Multivariable model	1.62 (1.31 to 2.01)	1.0	1.35 (1.08 to 1.67)
Patients without prior cardiovascular disease	1.53 (1.23 to 1.92)	1.0	1.27 (1.02 to 1.59)
Patients without deaths during the first 2 year of follow-up	2.48 (1.58 to 3.88)	1.0	1.82 (1.16 to 2.84)
Patients with follow-up period > = 24 months	1.85 (1.39 to 2.46)	1.0	1.50 (1.13 to 2.00)
Patients with age > = 18 years	1.62 (1.30 to 2.01)	1.0	1.33 (1.07 to 1.65)
Patients without beta-blocker	1.61 (1.23 to 2.10)	1.0	1.26 (1.06 to 1.66)
Patients without diuretics use	1.59 (1.26 to 2.01)	1.0	1.27 (1.02 to 1.61)
Patients without statin use	1.61 (1.28 to 2.03)	1.0	1.32 (1.05 to 1.66)

*Unless stated, model adjusted for age, sex, body mass index, current smoker, current alcohol use, systolic blood pressure, comorbidities, medication use, and laboratory variables. HR, hazards ratio

We confirmed this association with a sub-distribution hazard model. Compared with the moderate group, the adjusted HRs of all-cause mortality were 1.62 (95% CI, 1.31 to 2.01) and 1.35 (95%CI, 1.08 to 1.67) for the low and high groups, and the adjusted HRs of cardiovascular mortality were 1.74 (95% CI, 1.28 to 2.37) and 1.38 (95%CI, 1.12 to 1.88) for the two groups, respectively (multivariate model in Supplemental Tables 1 and 2).

### Sensitivity analysis

We performed sensitivity analyses in patients without prior cardiovascular disease, without deaths during the first 2 years of follow-up, the follow-up period > = 24 months, or age > = 18 years, respectively. Similar results were observed in those patients (Tables 3 and 4; Supplemental Tables 1 and 2). Notably, patients without deaths during the first 2 years of follow-up had higher adjusted HRs of all-cause and cardiovascular mortality (Tables 3 and 4; Supplemental Tables 1 and 2). Similar trends were observed in patients without beta-blocker, diuretics use, and statin use after considering the effect of medication use on total cholesterol.

**Table 4:** Association between total cholesterol and cardiovascular mortality*

	HR (95% CI) by total cholesterol		
	Low (< 4.10 mmol/L)	(Moderate 4.10 to 4.50 mmol/L)	High (> 4.50 mmol/L)
Univariate model	1.66 (1.23 to 2.25)	1.0	1.39 (1.12 to 1.89)
Multivariable model	1.72 (1.27 to 2.34)	1.0	1.38 (1.09 to 1.87)
Patients without prior cardiovascular disease	1.65 (1.20 to 2.27)	1.0	1.34 (1.06 to 1.85)
Patients without deaths during the first 2 year of follow-up	4.79 (2.19 to 10.47)	1.0	2.56 (1.16 to 5.64)
Patients with follow-up period > = 24 months	2.25 (1.44 to 3.50)	1.0	1.98 (1.27 to 3.07)
Patients with age > = 18 years	1.73 (1.27 to 2.35)	1.0	1.37 (1.06 to 1.87)
Patients without beta-blocker	1.53 (1.05 to 2.23)	1.0	1.23 (1.04 to 1.81)
Patients without diuretics use	1.70 (1.22 to 2.37)	1.0	1.28 (1.09 to 1.79)
Patients without statin use	1.57 (1.14 to 2.16)	1.0	1.29 (1.09 to 1.78)

*Unless stated, model adjusted for age, sex, body mass index, current smoker, current alcohol use, systolic blood pressure, comorbidities, medication use, and laboratory variables. HR, hazards ratio

### Subgroup analyses

To evaluate the modification effects of subgroups on the association between total cholesterol and mortality, we conducted the following subgroup analyses: age (< 65 or > = 65 years old), sex (male or female), diabetes mellitus (with or without), prior cardiovascular disease (with or without), hypertension (with or without), malnutrition (albumin < 36.0 or > = 36.0 g/L). P values for interactions were > 0.05 for all subgroups, suggesting that the increased risk of mortality associated with lower or higher total cholesterol was evident regardless of these factors (supplemental Tables 3 and 4).

## Discussion

Our data showed a U-shaped relationship of total cholesterol at the start of PD with all-cause and cardiovascular mortality in CAPD patients. The optimal range of total cholesterol, associated with the lowest mortality risk, was from 4.10 to 4.50 mmol/L (158.5 to 174.0 mg/dL). Our results were robust because similar trends were observed in various sensitivity and subgroup analyses. More than two decades ago, a study of more than 12,000 hemodialysis patients reported that all-cause mortality risk was significantly lower at higher total cholesterol levels [6]. Since then, many prospective studies in hemodialysis patients have replicated this inverse association [15, 16]. A similar inverse association was observed among PD patients. The paradoxical association of total cholesterol with mortality arouses some critical questions. Are there different biological effects of total cholesterol in dialysis patients compared with the general population? Should patients be advised to increase their nutrient intake to increase their cholesterol levels? Should new standards be considered for their lipid management [8]? Or is there an optimal range of total cholesterol associated with a lower risk of mortality in dialysis patients? Unfortunately, these issues above remain to be resolved. Another prospective study of 1191 incident dialysis patients reported that the reverse association between total cholesterol and mortality in dialysis patients was short-termed due to the time discrepancy of competing risks [3]. They stated that hypercholesterolemia was beneficial only in the short term but worsened survival over a long-term interval. Thus, this phenomenon has many potential explanations. A population-based cohort study from Taiwan reported that among 8032 PD patients, those without renal creatine clearance who had lower total cholesterol levels had higher mortality. The association was not observed among those with renal creatinine clearance who had lower total cholesterol levels [17]. They stated that at the initiation of dialysis, it was plausible to manage dyslipidemia as an extension of lipid-lowering therapy in advanced chronic kidney disease patients who were not dialysis-dependent but not in dialysis-dependent patients without renal creatinine clearance. We found a relatively narrow range of total cholesterol (158.5 to 174.0 mg/dL) in the present study, associated with the lowest mortality risk. Our findings suggested that too high or lower total cholesterol levels were associated with higher mortality risks. Maintaining the optimal range of total cholesterol may improve outcomes for CAPD patients. Nonetheless, total cholesterol levels in clinical practice are challenging to keep in a relatively narrow range for long periods. A large prospective multicenter study managing total cholesterol needs to be conducted to verify our results in CAPD patients.

Low albumin levels, a marker of inflammation and malnutrition, have increased susceptibility to death in dialysis patients [18]. An earlier study of 823 dialysis patients (including 125 PD patients) reported hypercholesterolemia is a risk factor for all-cause and cardiovascular mortality. This association was strengthened among patients with inflammation and/or malnutrition [8]. This study reported an inverse association of cholesterol levels with all-cause mortality, and in inflammation/malnutrition, a U-shaped relationship with cardiovascular mortality was found. Meanwhile, in patients without inflammation/ malnutrition, they found a strong, graded, positive association of total cholesterol with all-cause and cardiovascular mortality. In the present study, we did not find that inflammation and/or malnutrition affected the association of total cholesterol with all-cause and cardiovascular mortality. The inconsistent results may be that (1) different dialysis patients; (2) blood specimens in the earlier study were drawn at a median of 5.0 months from the initiation of dialysis, representing a stable status of dialysis. However, laboratory variables in our study were collected one week (5.3 ± 1.2 days) before the start of PD, representing a severe uremia status.

Large-scale multicenter long-term follow-up real-world study evaluating the effect of total cholesterol on all-cause mortality and cardiovascular mortality were clear strengths of our study. The large sample size ensures statistical efficiency. In addition, we conducted various sensitivity analyses, including the potential for reverse causation, the effect of medications on total cholesterol, and fully observing endpoints. Nonetheless, the present study has several limitations. First, based on a retrospective study, we were unable to draw a causal relationship between total cholesterol and mortality, and the possibility of residual confounding may arise from unmeasured variables. Second, we failed to repeatedly check total cholesterol during follow-up, underestimating the association between total cholesterol levels and mortality [19]. Third, missing values were replaced by the most recent available deals instead of using multiple imputations. Although multiple imputations can fill in missing values at random, the latest available value may more appropriately represent the patient’s clinical status.

In conclusion, at the start of PD, total cholesterol levels between 4.10 and 4.50 mmol/L (158.5 to 174.0 mg/dL), as an optimal range, were associated with the lowest risk of death than higher or lower levels. Our findings suggested that further studies altering pre-dialysis cholesterol and assessing outcomes prospectively needed to be conducted. We know statin studies in dialysis have not been positive. As with all such studies, it is hypothesis-generating. Overall, once these issues are dealt with, the manuscript is a useful addition to the scientific literature.

## Electronic supplementary material

Below is the link to the electronic supplementary material.

Supplementary Material 1

## Data Availability

All data generated or analysed during this study are included in this published articleand its supplementary information files.
