# Supplementary material for: Total cholesterol and mortality in peritoneal dialysis: a retrospective cohort study
Source: BMC Nephrol. 2023 May 23;24:142. doi: 10.1186/s12882-023-03187-1 (PMC10207729; doi:10.1186/s12882-023-03187-1)
Supplement: Supplementary file 1 — Supplementary Material 1 [file 12882_2023_3187_MOESM1_ESM.docx]

**Supplementary material**

**Total Cholesterol and Mortality in Peritoneal Dialysis**

**Junnan Wu ^1†^, Ruifeng Yang^2†^, Xiaoyang Wang ^3^, Xiaojiang Zhan ^4^, Yueqiang Wen ^5^, Xiaoran Feng ^6^, Niansong Wang ^2, 7^, Fenfen Peng ^8^, Guihua Jian^2*^, Xianfeng Wu ^2,7*^**

^1^ Department of Nephrology, Zhejiang University Medical College Affiliated Sir Run Run Shaw Hospital, HangZhou, China

^2^ Department of Nephrology, Shanghai Sixth People's Hospital Affiliated to Shanghai Jiao Tong University School of Medicine, Shanghai, China.

^3^ Department of Nephrology, The First Affiliated Hospital of Zhengzhou University, Zhengzhou, China.

^4^ Department of Nephrology, The First Affiliated Hospital of Nanchang University, Nanchang, China.

^5^ Department of Nephrology, The Second Affiliated Hospital of Guangzhou Medical University, Guangzhou, China.

^6^ Department of Nephrology, Jiujiang No. 1 People’s Hospital, Jiujiang, China.

^7^ Clinical Research Center for Chronic Kidney Disease, Shanghai Sixth People's Hospital Affiliated to Shanghai Jiao Tong University School of Medicine, Shanghai, China

^8^ Department of Nephrology, Zhujiang Hospital of Southern Medical University, Guangzhou, China.

†These authors have contributed equally to this work and share the first authorship.

**^*^****Correspondence:**

Guihua Jian,

Department of Nephrology, Shanghai Sixth People's Hospital Affiliated to Shanghai Jiao Tong University School of Medicine, No.600, Yi Shan Road, Shanghai, 200233, China,

Tel: 0086-021-64369181,

E-mail: [1433363218@qq.com](mailto:1433363218@qq.com)

Xianfeng Wu,

Department of Nephrology/Clinical Research Center for Chronic Kidney Disease, Shanghai Sixth People's Hospital Affiliated to Shanghai Jiao Tong University School of Medicine, No.600, Yi Shan Road, Shanghai, 200233, China,

Tel: 0086-021-64369181,

E-mail: [xianfengwu2@163.com](mailto:xianfengwu2@163.com)

**Materials & Methods**

**Study Design and Participants**

***Peritoneal dialysis centers of five tertiary hospitals***

The First Affiliated Hospital of Zhengzhou University, Zhengzhou, China.

The First Affiliated Hospital of Nanchang University, Nanchang, China.

Jiujiang No. 1 People’s Hospital, Jiujiang, China.

Zhujiang Hospital of Southern Medical University, Guangzhou, China.

The Second Affiliated Hospital of Guangzhou Medical University, Guangzhou, China.

***Data Collection***

Patients were advised to initiate peritoneal dialysis (PD) with professional and clinical evaluation from nephrologists. In all patients, thorough medical records were reviewed by trained nurses in each dialysis center at study entry. In China, the patient must receive the first dialysis in the hospital, suggesting most of the patient's data can be obtained within one week before the first dialysis. Thus, we defined baseline as one week (5.3±1.2 days) before the first continuous ambulatory peritoneal dialysis (CAPD). All laboratory parameters from fasting blood samples were measured in each tertiary hospital's laboratory department.

***Dialysis procedure***

All patients received CAPD treatment. Conventional dialysis solutions (Dianeal 1.5%, 2.5%, or 4.25% dextrose; Baxter Healthcare, Guangzhou, China), Y sets, and twin bag systems were used in all CAPD patients. No patients received automated PD.

***Follow-up***

There was no exposure to all patients with any intervention. Patients needed to return to each center at least quarterly for an overall medical assessment. The trained nurses conducted monthly face-to-face interviews or monthly telephone interviews to assess their general condition and related medications.

***Outcome measurements***

We determined death causes based on medical files of admission. If patients died out of hospitals, we determined death causes according to interviewing with family members by telephone to acknowledge death's circumstances, combining with information from medical records of peritoneal dialysis centers.

***Definitions***

Cardiovascular mortality included death associated with an acute myocardial ischemic event, heart failure, hemorrhagic or thromboembolic stroke, malignant arrhythmia, and sudden cardiac death, based on the International Classification of Diseases Clinical Modification, 9th Revision. Sudden cardiac death is defined as unexpected, nontraumatic death occurring within one hour of the onset of new or worsening symptoms (witnessed arrest) or, if unwitnessed, within 24 hours of last being seen alive[1]. Hypertension was defined as systolic blood pressure > 140 mmHg, diastolic blood pressure > 90 mmHg, or the use of antihypertensive medications. Diabetes mellitus was defined as a history of diabetes mellitus. Current smoking was defined as at least one cigarette a day, and current alcohol consumption was defined as > 20 g of ethanol a day[2]. The Chronic Kidney Disease Epidemiology Collaboration equation was used to calculate eGFR[3].

**Missing data**

In China, the patient must receive the first dialysis procedure in the hospital. Thus, hospitalization records of the first dialysis procedure were routinely available. To obtain missing patients' demographic characteristics, comorbid conditions, medication use, and laboratory variables, we had checked the medical records of receiving the first PD procedure. The primary outcome was not missed.

**Figure S1. Cumulative mortality by categories of total cholesterol in multivariate analysis.**


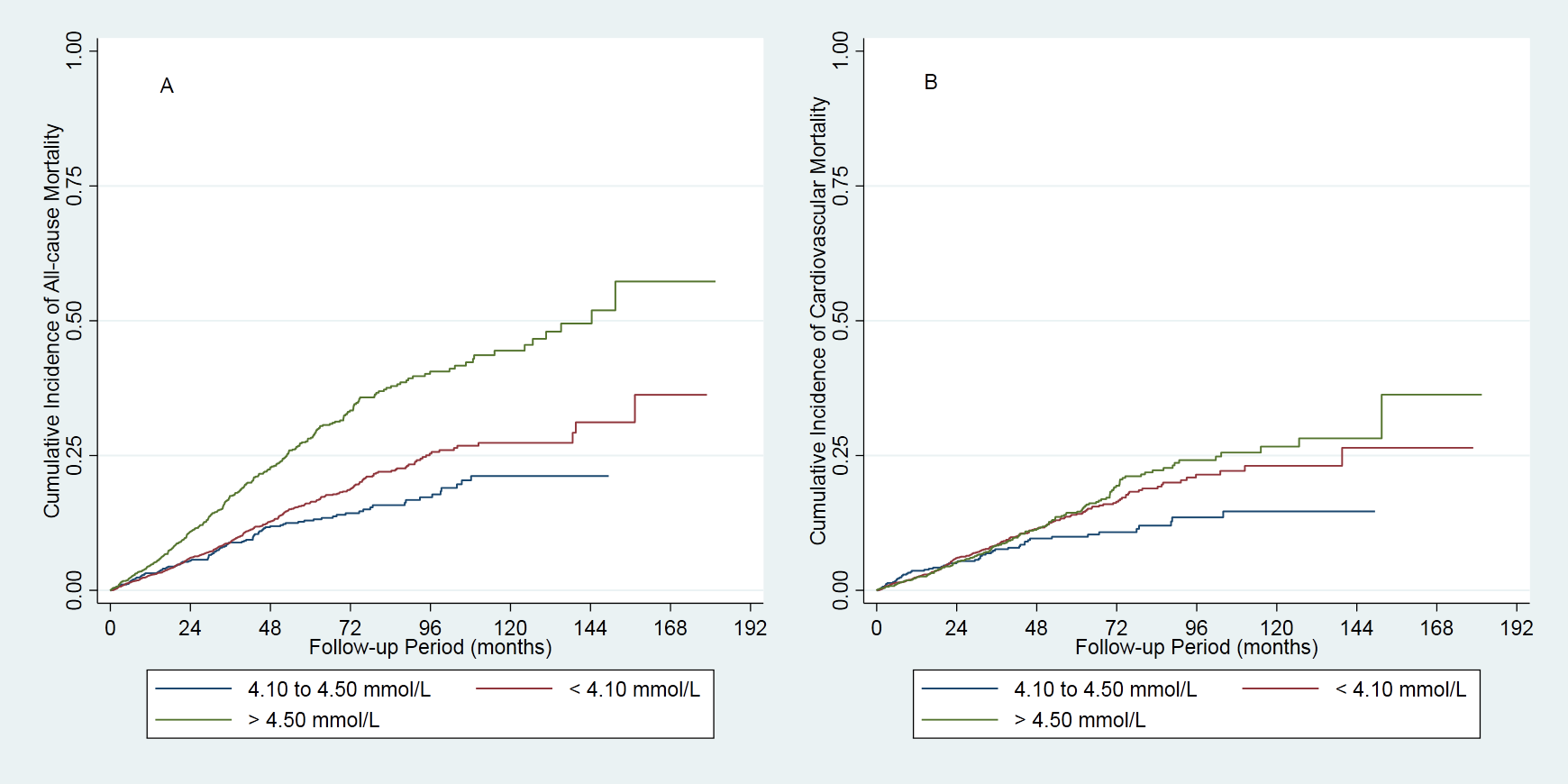


Panel A showed cumulative all-cause mortality by categories of total cholesterol. Panel B showed cumulative cardiovascular mortality by categories of total cholesterol. Adjusted for age, sex, body mass index, current smoker, current alcohol use, systolic blood pressure, comorbidities, medication use, and laboratory variables.

**Table S1. Association between total cholesterol and all-cause mortality using subdistribution hazard model ***

|  | HR (95% CI) by total cholesterol | | |
| --- | --- | --- | --- |
|  | Low (< 4.10 mmol/L) | Moderate (4.10 to 4.50 mmol/L) | High (> 4.50 mmol/L) |
| Univariate model | 1.58 (1.27 to 1.95) | 1.0 | 1.39 (1.12 to 1.72) |
| Multivariable model | 1.62 (1.31 to 2.01) | 1.0 | 1.35 (1.08 to 1.67) |
| Patients without prior cardiovascular disease | 1.53 (1.23 to 1.92) | 1.0 | 1.27 (1.02 to 1.59) |
| Patients without deaths during the first 2 years of follow-up | 2.28 (1.46 to 3.57) | 1.0 | 1.75 (1.12 to 2.74) |
| Patients with follow-up period >= 24 months | 1.74 (1.31 to 2.32) | 1.0 | 1.42 (1.07 to 1.90) |
| Patients with age >= 18 years | 1.62 (1.30 to 2.01) | 1.0 | 1.33 (1.07 to 1.65) |
| Patients without diuretics use | 1.59 (1.26 to 2.01) | 1.0 | 1.27 (1.02 to 1.61) |
| Patients without statin use | 1.61 (1.28 to 2.03) | 1.0 | 1.32 (1.05 to 1.66) |

*Unless stated, model adjusted for age, sex, body mass index, current smoker, current alcohol use, diabetes mellitus, prior cardiovascular disease, hypertension, albumin, eGFR, HDL-C, and LDL-C. HR, hazards ratio.

**Table S2. Association between total cholesterol and cardiovascular mortality using subdistribution hazard model ***

|  | HR (95% CI) by total cholesterol | | |
| --- | --- | --- | --- |
|  | Low (< 4.10 mmol/L) | Moderate (4.10 to 4.50 mmol/L) | High (> 4.50 mmol/L) |
| Univariate model | 1.68 (1.24 to 2.27) | 1.0 | 1.40 (1.13 to 1.90) |
| Multivariable model | 1.74 (1.28 to 2.37) | 1.0 | 1.38 (1.12 to 1.88) |
| Patients without prior cardiovascular disease | 1.66 (1.21 to 2.29) | 1.0 | 1.35 (1.07 to 1.86) |
| Patients without deaths during the first 2 years of follow-up | 4.38 (2.01 to 9.55) | 1.0 | 2.45 (1.11 to 5.41) |
| Patients with follow-up period >= 24 months | 2.13 (1.36 to 3.31) | 1.0 | 1.86 (1.20 to 2.89) |
| Patients with age >= 18 years | 1.74 (1.28 to 2.37) | 1.0 | 1.38 (1.10 to 1.87) |
| Patients without diuretics use | 1.72 (1.24 to 2.40) | 1.0 | 1.28 (1.02 to 1.79) |
| Patients without statin use | 1.58 (1.15 to 2.18) | 1.0 | 1.30 (1.04 to 1.80) |

* Unless stated, model adjusted for age, sex, body mass index, current smoker, current alcohol use, diabetes mellitus, prior cardiovascular disease, hypertension, albumin, eGFR, HDL-C, and LDL-C. HR, hazards ratio.

**Table S3. Association of total cholesterol with all-cause mortality in subgroups.**

|  | HR (95% CI) by total cholesterol | | |  |
| --- | --- | --- | --- | --- |
|  | Low (< 4.10 mmol/L) | Moderate (4.10 to 4.50 mmol/L) | High (> 4.50 mmol/L) | P-interaction |
| Age ≥65 years | 1.82 (1.10-3.00) | 1.0 | 1.32 (0.79-2.17) | 0.339 |
| Age <65 years | 1.60 (1.26-2.04) | 1.0 | 1.37 (1.08-1.74) |  |
| Male | 1.52 (1.13-2.05) | 1.0 | 1.40 (1.03-1.89) | 0.410 |
| Female | 1.74 (1.27-2.38) | 1.0 | 1.28 (0.94-1.75) |  |
| Diabetes mellitus | 1.54 (0.96-2.48) | 1.0 | 1.10 (0.69-1.75) | 0.408 |
| No diabetes mellitus | 1.66 (1.30-2.13) | 1.0 | 1.42 (1.11-1.82) |  |
| Prior cardiovascular disease | 2.98 (1.29-6.92) | 1.0 | 2.45 (1.09-5.49) | 0.175 |
| No prior cardiovascular disease | 1.53 (1.23-1.92) | 1.0 | 1.27 (1.02-1.59) |  |
| Hypertension | 1.64 (1.27-2.12) | 1.0 | 1.26 (0.97-1.64) | 0.399 |
| No hypertension | 1.50 (1.01-2.23) | 1.0 | 1.48 (1.01-2.18) |  |
| Albumin ≥36.0g/L | 2.04 (1.41-2.95) | 1.0 | 1.68 (1.15-2.46) | 0.908 |
| Albumin <36.0g/L | 1.35 (1.04-1.76) | 1.0 | 1.23 (0.94-1.59) |  |

All analyses adjusted for age, sex, body mass index, current smoker, current alcohol use, diabetes mellitus, prior cardiovascular disease, hypertension, albumin, eGFR, HDL-C, and LDL-C, except the subgroup variable. HR, hazards ratio.

**Table S4. Association of total cholesterol with cardiovascular mortality in subgroups.**

|  | HR (95% CI) by total cholesterol | | |  |
| --- | --- | --- | --- | --- |
|  | Low (< 4.10 mmol/L) | Moderate (4.10 to 4.50 mmol/L) | High (> 4.50 mmol/L) | P-interaction |
| ≥65 years | 1.63 (0.80-3.33) | 1.0 | 1.47 (0.73-2.97) | 0.644 |
| <65 years | 1.78 (1.26-2.50) | 1.0 | 1.37 (0.97-1.93) |  |
| Male | 1.64 (1.08-2.47) | 1.0 | 1.45 (0.96-2.20) | 0.370 |
| Female | 1.88 (1.18-2.98) | 1.0 | 1.27 (0.80-2.02) |  |
| Diabetes mellitus | 2.13 (1.09-4.16) | 1.0 | 1.43 (0.74-2.73) | 0.870 |
| No diabetes mellitus | 1.64 (1.16-2.33) | 1.0 | 1.33 (0.93-1.90) |  |
| Prior cardiovascular disease | 2.93 (0.96-8.92) | 1.0 | 1.83 (0.61-5.47) | 0.907 |
| No prior cardiovascular disease | 1.65 (1.20-2.27) | 1.0 | 1.34 (1.06-1.85) |  |
| Hypertension | 2.00 (1.36-2.94) | 1.0 | 1.45 (0.98-2.15) | 0.794 |
| No hypertension | 1.20 (0.71-2.01) | 1.0 | 1.12 (0.67-1.87) |  |
| Albumin ≥36.0g/L | 1.86 (1.15-3.00) | 1.0 | 1.34 (0.81-2.21) | 0.803 |
| Albumin <36.0g/L | 1.58 (1.06-2.33) | 1.0 | 1.38 (0.93-2.03) |  |

All analyses adjusted for age, sex, body mass index, current smoker, current alcohol use, diabetes mellitus, prior cardiovascular disease, hypertension, albumin, eGFR, HDL-C, and LDL-C, except the subgroup variable. HR, hazards ratio.

**Reference**

1. European Heart Rhythm A, Heart Rhythm S, Zipes DP, Camm AJ, Borggrefe M, Buxton AE, Chaitman B, Fromer M, Gregoratos G, Klein G *et al*: **ACC/AHA/ESC 2006 guidelines for management of patients with ventricular arrhythmias and the prevention of sudden cardiac death: a report of the American College of Cardiology/American Heart Association Task Force and the European Society of Cardiology Committee for Practice Guidelines (Writing Committee to Develop Guidelines for Management of Patients With Ventricular Arrhythmias and the Prevention of Sudden Cardiac Death)**. *J Am Coll Cardiol* 2006, **48**(5):e247-346.

2. Tu W, Wu J, Jian G, Lori J, Tang Y, Cheng H, Wu X, Wang N: **Asymptomatic hyperuricemia and incident stroke in elderly Chinese patients without comorbidities**. *Eur J Clin Nutr* 2019, **73**(10):1392-1402.

3. Zhang L, Wang F, Wang L, Wang W, Liu B, Liu J, Chen M, He Q, Liao Y, Yu X *et al*: **Prevalence of chronic kidney disease in China: a cross-sectional survey**. *Lancet* 2012, **379**(9818):815-822.
